# Correlated Expression of Notch2 and ADAM17 in Primary Sjögren’s Syndrome Salivary Glands

**DOI:** 10.3390/jcm15010182

**Published:** 2025-12-26

**Authors:** Margherita Sisto, Sabrina Lisi, Roberto Tamma, Michelina De Giorgis, Giuseppe Ingravallo, Mario Della Mura, Joana Sorino, Eliano Cascardi, Domenico Ribatti

**Affiliations:** 1 Department of Translational Biomedicine and Neuroscience (DiBraiN), Section of Human Anatomy and Histology, University of Bari “Aldo Moro”, Piazza Giulio Cesare 1, 70124 Bari, Italydomenico.ribatti@uniba.it (D.R.); 2 Department of Precision and Regenerative Medicine and Ionian Area (DiMePRe-J), Section of Molecular Pathology, University of Bari “Aldo Moro”, 70124 Bari, Italy

**Keywords:** primary Sjögren’s syndrome, Notch2, ADAM17, inflammation

## Abstract

**Background/Objectives**: The Notch–ADAM17 pathway is a fundamental signaling mechanism where ADAM17, a disintegrin and metalloprotease, cleaves the Notch receptor after the Notch receptor binds to a ligand. Crosstalk between Notch and ADAM17 is often altered in pathological situations. Alterations in Notch2 expression, in particular, appears to be correlated with the onset of various autoimmune diseases. In primary Sjögren’s disease (pSjD), an autoimmune disorder characterized by chronic inflammation, the role of ADAM17 has been extensively explored, but a correlation with Notch2 has not yet been evaluated. **Methods**: To analyze the gene and protein expression of Notch2 in pSjD and a possible correlation with ADAM17 expression and with the patient’s inflammatory grade, we employed an integrated co-detection protocol to analyze salivary gland tissue sections by combining in situ hybridization (ISH) with immunohistochemistry (IHC). **Results**: combined ISH/IHC allows us to demonstrate an increased expression of Notch2 mRNA and protein in pSjD salivary glands (SGs) biopsies, which appears correlated with an increased expression of ADAM17, both in acinar and duct cells and in infiltrating lymphocytes. Notch2/ADAM17 expression is higher in biopsies of pSjD SGs characterized by a high degree of inflammation. **Conclusions**: this work demonstrates the correlated expression in pSjD SGs of ADAM17, which plays multiple roles in the pathogenesis of SjD, and Notch2, widely considered a key player in various inflammatory mechanisms, offering a starting point for future therapeutic interventions to investigate.

## 1. Introduction

**Figure 1:**
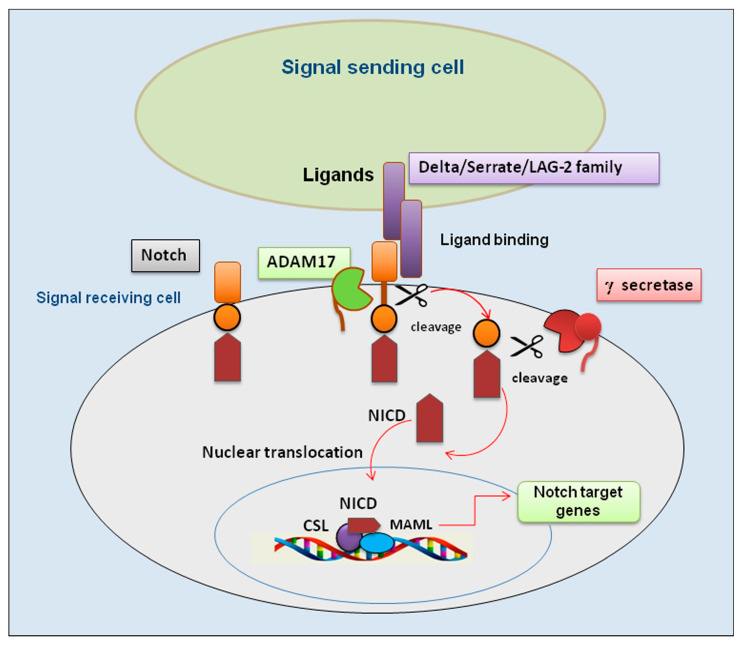
An overview of the Notch/ADAM17 signaling pathway. Representation of key events involved in activation of the Notch/ADAM17 pathway. Binding of ligands by a signal-sending cell leads the Notch receptor to a conformational change that undergoes the first proteolytic cleavage by ADAM17; subsequently, a second proteolytic cleavage by γ-secretase occurs that permits a nuclear translocation of the remaining NICD. This process leads to the association with the transcription factor CSL and transcriptional co-activator MAML to form a transcriptional activation complex. ADAM 17 (A Disintegrin And Metalloproteinase); CSL (suppressor of hairless); DLL4 (delta-like ligand 4); JAG (jagged); MAML (mastermind-like); NICD (Notch intracellular domain).

The Notch pathway is an evolutionarily preserved signaling system that modulates multifaceted activities in a wide range of cellular events, which is implicated in reorganization and regeneration of tissues and in intricate interactions with other signaling pathways [1,2]. The induction of Notch signaling occurs through a finely regulated cascade of proteolytic cleavages with subsequent triggering of downstream target genes [3,4]. Notch receptors can cooperate with Notch ligands when a ligand of the Delta/Serrate/LAG-2 family (positioned on the surface of bordering cells) binds to the extracellular domain of the Notch receptor and activates proteolytic cleavage by a member of the disintegrin and metalloprotease (ADAM) family. ADAM10 and/or ADAM17 cleavage produces a substrate for an additional cleavage, generating a Notch extracellular form of the receptor, which is a membrane-bound Notch fragment. Following, the presenilin-containing γ-secretase complex cleaves the Notch receptor, leading to the formation of the Notch intracellular domain (NICD), which is released and corresponds to the activated form of Notch. Moreover, NICD translocates to the nucleus and forms complexes with specific DNA-binding proteins (CBF1/Suppressor of Hairless/LAG-1 and Mastermind/SEL-8) and transcriptionally activates target genes [3]. A schematic representation of the interrelationship between Notch and ADAM17 is shown in Figure 1.

Notch2 is one of the four receptors (Notch1–4) commonly expressed in a variety of cancer cells, including gastric, hematological, and lung cancer. In this context, Notch2 is the main factor activating alveolar morphogenesis and maintaining airway epithelial integrity [5]. Interestingly, Notch2 enhances the activity of pathways associated with inflammation through the regulation of immunomodulatory functions by CD4+ T cells [6]. Therefore, Notch2 has been shown to be upregulated in inflammatory states that characterize diseases like rheumatoid arthritis (RA) and bacterial and viral infections [6,7,8,9]. Not surprisingly, recent evidence points to Notch2 signaling as an important player in autoimmunity, characterized by sustained and chronic inflammation [10]. Effectively, aberrant Notch2 signaling has been observed in clinical samples of patients affected by RA [11,12,13,14,15,16,17], systemic lupus erythematosus (SLE) [18,19], and systemic sclerosis [20].

Currently, a possible involvement of an altered Notch2 expression in the pathogenesis of Sjögren’s syndrome disease (SjD) has not yet been experimentally analyzed. SjD is a complex, disabling systemic autoimmune disorder affecting several tissues. Patients have, as a primary characteristic, altered tissue organization and consequently a reduced functionality of the exocrine glands, which manifests primarily in the salivary glands (SGs) and lacrimal glands, representing the first target of inflammation [21]. Over the past decade, major advances have been made in understanding the pathogenesis of primary SjD (pSjD), so called because it is not associated with secondary pathologies, and the trajectory of research is swiftly advancing, with a focused lens on the role of Notch2 in pSjD.

Most of our knowledge of the pathogenesis of SGs from pSjD patients relies on approaches using standard gene and protein analysis methods. It is crucial to unbiasedly understand the molecular changes in the SGs at the cellular level, within the tissue context. Due to the multiple benefits of both in situ hybridization (ISH) and immunohistochemistry (IHC) to detect gene and protein expression and the robustness of the RNAscope assay, in this study, we employed a dual ISH–IHC protocol to combine both assays on the same biopsy specimens of SGs from pSjD expressing an increasing degree of inflammation. This method is called dual RNAscope ISH–IHC and allows us to simultaneously detect mRNA and protein expression in formalin-fixed, paraffin-embedded tissue sections [22]. We analyzed Notch2 expression at both the gene and protein levels and a possible correlation with ADAM17 expression in the same biopsy specimens. The objective was to demonstrate the hypothetical correlation between Notch2 and ADAM17 expression in pSjD, which could be more detectable in patients with a high degree of inflammation of the SGs. This would lead to the identification of new therapeutic molecular targets and the discovery of new pathways that determine the loss of glandular function.

## 2. Materials and Methods

### 2.1. Patients, Clinical Specimens

A subset of consecutive labial minor salivary glands (MSGs) stained only with hematoxylin and eosin (H&E) was randomly retrieved from the archive of the Anatomical and Molecular Pathology Section, School of Medicine, University of Bari, Italy. Biopsies were performed between 2022 and the end of 2023 in individuals with a clinical suspicion of pSjD for diagnostic purposes only and after signing a written informed consent. The biopsies were fixed in formalin and paraffin (FFP) embedded. This study was approved by the local ethical review committee, and the experiments were conducted according to the tenets of the Declaration of Helsinki. Labial MSGs biopsy samples were taken from the suspected patients with SjD from the lower lip under local anesthesia through normal mucosa, according to the explant outgrowth technique [23]. Bioptic sections of MSGs belonging to different groups of patients with several grades of inflammation were identified. The department selected 34 biopsies from which bioptic specimens belonging to the same number of patients (n = 10) were chosen, excluding dubious cases (n = 4). Healthy control subjects (n = 10) were analyzed for an abnormal salivary function and suspected pSjD, which resulted in a normal biopsy. According to the updated 2002 American-European criteria, each patient had a diagnosed illness [24]. A positive Schirmer’s test (less than 5 mm wetting of a strip of filter paper per five minutes (min)), Rose Bengal staining (increased uptake of Rose Bengal dye in devitalized areas in the conjunctiva and cornea), and the presence of at least one of the following autoantibodies, anti-Ro/SSA, anti-La/SSB, anti-nuclear antibodies, and rheumatoid factor, were present in every patient.

None of the patients studied had received any glucocorticoid and/or immunosuppressive drug treatment until biopsy performance. Moreover, since assessments of FS and GC are semi-quantitative methods where the histopathological tissue architecture might differ on multiple sections taken from the same gland, a re-evaluation of the FS and GC was conducted on MSGs tissue from all pSjD patients in order to eliminate potential discrepancies. This was performed on hematoxylin–eosin-stained sections in accordance with our previous laboratory examinations of such structures. Patient stratification was carried out by stratifying the pSjD patients into three distinct groups, where each group represented a disease stage according to the degree of inflammation in their MSG using the inflammatory severity index. Patients with low lesions (FS ≤ 1) were included in the first group (S1), patients with moderate lesions (FS ≥ 2) were included in the second group (S2), while patients displaying severe lesions and GC in the MSG tissue (FS ≥ 2 and GC+) were classified in the third group (S3). At the time of biopsy, the median age of subjects in the control group was 49.8 years (range 21–66), the median age of patients in the group with low focus scores was 57.7 years (range 24.6–66.9), the median age of patients in the group with intermediate focus scores was 52.4 years (range 24.8–66.7), and the median age of patients in the group with high focus scores was 52.9 years (range 38.9–70.1). The clinical characteristics of pSjD patients and healthy controls are summarized in Table 1.

### 2.2. Notch2 mRNA ISH Assay

An RNAscope assay was performed on FFPE biopsies using an RNAscope 2.5 HD Reagent Kit (RED 322350, Advanced Cell Diagnostics (ACD), Hayward, CA, USA). Briefly, tissue sections were deparaffinized with xylene and 100% ethanol and incubated with pretreat-1 solution for 10 min., pretreat-2 for 15 min., and pretreat-3 for 30 min (Pretreatment kit 322330, ACD). The slides were then hybridized with a probe, Hs-NOTCH2 (ref. 488101), positive control probe, Hs-PPIB (ref. 313901), and negative control probe, DapB (ref. 310043) in the HybEZ oven (ACD) at 40 °C for 2 h. The Hs-PPIB probe for human housekeeping gene PPIB was used as a control to ensure RNA quality. After hybridizations, slides were subjected to signal amplification using an HD 2.5 detection Kit, and a hybridization signal was detected using a mixture of Fast-RED solutions A and B (1:60). Following counterstaining with Gill’s hematoxylin, the slides were dried in a dry oven at 60 °C for 15 min and subsequently mounted using Glycergel Mounting Medium (Dako, C0563). Sections from each experimental group were scanned, utilizing the whole-slide morphometric analysis scanning platform of the Aperio Scanscope CS (Leica Biosystems, Nussloch, Germany). All slides were scanned at the highest available magnification (40×) and saved as digital high-resolution images on the workstation linked to the instrument. According to the PPIB evaluation, all cases were included in the analysis. Digital slides were assessed using Aperio ImageScope v.11 software (Leica Biosystems, Nussloch, Germany) at 20× magnification, with ten fields of equal area selected for analysis at 40× magnification. The mRNA expression was evaluated using the Aperio RNA ISH algorithm, which provides standardized quantification of RNA ISH staining in whole slide images of FFPE tissue. This algorithm automatically quantifies the staining across entire slides, counting individual molecular signals and clusters within the cells. The results obtained are categorized into three ranges: 1+ for cells containing 2 to 5 dots; 2+ for cells containing 6 to 20 dots; and 3+ for cells containing more than 20 dots. The statistical significance of the differences between the mean values of the percent labeled areas for ABC and GCB tumor specimens was determined using the 2-way ANOVA test in GraphPad Prism 5.0 software (GraphPad Software, La Jolla, CA, USA). Findings were deemed significant at *p* values < 0.05.

### 2.3. Notch2 Protein IHC

Serial 3 μm sections of healthy and pSjD formalin-fixed, paraffin-embedded minor SGs tissues were used for immunohistochemical staining. Paraffin sections were deparaffinized with xylene and hydrated with a series of graded ethanol washes. After deparaffinization and dehydration, the slides were washed in phosphate-buffered saline (PBS) (pH 7.6 3 × 10 min) then immersed in EDTA buffer (0.01 M, pH 8.0) for 20 min in a water bath at 98 °C to unmask antigens. The sections were immunolabeled according to the following procedure: blockade of endogenous peroxidase by treatment with 3% hydrogen peroxide solution in water for 10 min at room temperature (RT); rinsing for 3 × 10 min in PBS, pH 7.6; preincubation in non-immune donkey serum (Dako LSAB Kit, Dako, CA, USA) for 1 h at RT; and incubation overnight at 4 °C with primary anti-Notch2 Antibody (Ab) (Cell Signaling; 1:100 dilution). The slides were washed for 3 × 10 min in PBS and then incubated with the secondary Ab (Santa Cruz Biotechnology, Santa Cruz, TX, USA) diluted 1:200 in PBS for 1 h at RT, rinsed for 3 × 10 min in PBS, incubated with the streptavidin–peroxidase complex (Vector Laboratories, Mowry Ave, Newark, CA, USA) for 1 h at RT, incubated with the chromogen 3,3-diaminobenzidine tetrahydrochloride (DAB) (Vector Laboratories) for 10 min at RT, then counterstained with hematoxylin (Merck Eurolab, Dietikon, Switzerland). Negative controls of the immunoreactions were performed by replacing the primary Ab with donkey serum diluted 1:10 in PBS. After the addition of the secondary Ab, no specific immunostaining was observed in the negative controls.

### 2.4. ADAM17 Protein IHC

The slides used in the RNAscope assay have been disassembled by incubation at 60 °C in an oven for a few minutes. Afterwards, slides were washed in PBS and processed by the IHC classical protocol. Sections were pre-treated with sodium citrate pH 6.1 or pH 9 (Dako Corporation, Milan, Italy) in Dako PT Link for antigen retrieval solution for 30 min at 98 °C and then incubated with rabbit polyclonal anti-ADAM17 antibody (GTX101358, GeneTex International Corporation, Alton Pkwy, Irvine, CA, USA), diluted 1:250. After incubation with the relative secondary Ab (Santa Cruz Biotechnology, Dallas, TX, USA) diluted 1: 200 in PBS for 1 h at RT, the streptavidin–peroxidase complex (Vector Laboratories, Newark, CA, USA) was added before the incubation with DAB for 10 min at RT. Thereafter, the sections were counterstained with Mayer hematoxylin and mounted in synthetic medium.

### 2.5. IHC Analysis and Quantification

The protein expression was assessed with the Positive Pixel Count algorithm embedded in the Aperio ImageScope software, version 12.3, and reported as positivity percentage, defined as the number of positively stained pixels on the total pixels in the image. Sections from each experimental group (n.10), 10 cases per group, were scanned using the whole-slide morphometric analysis scanning platform of the Aperio Scanscope CS (Leica Biosystems, Nussloch, Germany). All the slides were scanned at the maximum available magnification (40×) and stored as digital high-resolution images on the workstation associated with the instrument. Digital slides were inspected with Aperio ImageScope v.11 software (Leica Biosystems, Nussloch, Germany) at 20× magnification, and ten fields with an equal area were selected for the analysis at 40× magnification.

The statistical significance of differences between the mean values of the percent labeled areas between pSjD SGs specimens and control tissues was determined by the 2-way Anova test in GraphPad Prism 5.0 software (GraphPad software, La Jolla, CA, USA). Findings were considered significant at *p* values < 0.05.

### 2.6. Aperio Digital ISH and IHC Analysis and Quantification

High-resolution digital scans of stained tissue were acquired using an Aperio Scanscope CS2 (Leica Biosystems, Nussloch, Germany), resulting in the creation of a digital archive of these high-resolution images. The digital slides were examined with Aperio ImageScope v.11 software (Leica Biosystems) at a magnification of 10×, from which ten fields of equal area were randomly chosen for analysis at 40× magnification. The expression levels of Notch2 mRNA and Notch2 protein were evaluated using the Positive Pixel Count algorithm integrated within the Aperio ImageScope software, with results reported as a percentage of positivity, which is defined as the ratio of positively stained pixels to the total number of pixels in the image. This methodology facilitates a dependable automatic assessment of the staining extent in the tissue while also minimizing the variability linked to human error.

### 2.7. Statistic

The normalized data for mRNA and protein expression were analyzed to determine mean values ± standard error (s.e.). The differences among the parameters were assessed using Student’s *t*-test. Spearman’s correlation coefficients were computed to assess the relationships between Notch2, ADAM17, and the pSjD inflammatory grade. A *p*-value of less than 0.05 was considered statistically significant.

## 3. Results

### 3.1. SjD Histopathology of the Bioptic SGs Specimens

**Figure 2:**
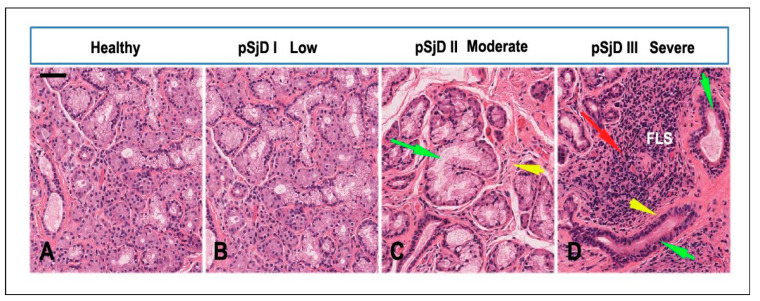
Histopathological analysis of the minor salivary gland biopsies in patients with pSjD stained with hematoxylin and eosin. (**A**) Normal SGs tissue, (**B**–**D**) pSjD bioptic tissues with low (pSjD I), moderate (pSjD II), and severe (pSjD III) inflammatory grades. Red Arrow: focal lymphocytic sialadenitis (FLS) with perivascular or periductular aggregates of lymphocytes. Green arrows: dilated ducts of the minor SGs biopsy. Yellow arrows: interstitial fibrosis. pSjD (primary Sjögren’s disease) BAR: 20 μm.

The glandular histopathology of the three inflammatory grades of SjD patients, low, intermediate, and severe, was compared relatively to healthy subjects (Figure 2A–D). The histopathological features of SGs in SjD include parenchymal and ductal changes. It was possible to see, in all the biopsies used, a decrease in or even disappearance of acini, lymphocyte infiltration and proliferation of the lining cells, and formation of myoepithelial cell islands, which appear to be more pronounced in the severe grade of inflammation (panel D). Furthermore, focal inflammation in SjD SGs tissue, especially in severe cases (pSjD III), is accompanied by acinar atrophy, ductal dilatation, and fibrosis. The presence of adipose tissue is noticeable, and the lobular fibrosis is also related to the severity of inflammation (arrows in the Figure 2B–D).

### 3.2. Notch2 mRNA Detection in SjD SGs by ISH

**Figure 3:**
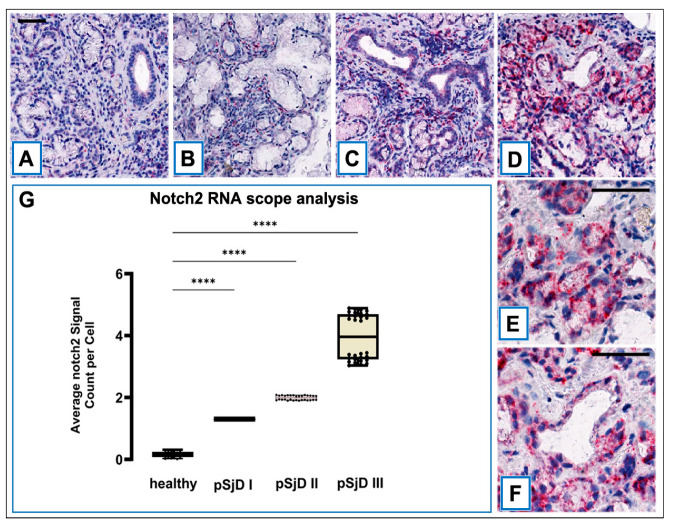
RNAscope assay in situ hybridization technique was performed in order to evaluate *Notch2* mRNA in pSjD SGs tissue sections of healthy control and patients with different inflammatory grades (pSjDI, pSjDII, pSjDIII) (**A**–**D**). It notes the presence of dotted positive red *Notch2* mRNA signals in the epithelial cells of the acini and ducts, as well as in the interlobular infiltrative cells (**B**–**D**). Images (**E**,**F**) represent the magnification of acini (**E**) and ducts (**F**) of pSjD III tissues. In particular, it observes the presence of numerous clustered red dot signals in the acini, ducts, and infiltrate cells in pSjD III. All images were scanned and analyzed with an Aperio ImageScope instrument. Image (**G**) represents the quantitation of RNA ISH staining of *Notch2* mRNA positivity in healthy and pSjD (I, II, III) expressed in terms of average of positive signal count per cell; the graph demonstrates a significant increase in *Notch2* mRNA in the different inflammatory grades of pSjD (**** *p* < 0 01) (data represent mean ± SE of three independent experiments). BAR = 20 μm.

RNA ISH was performed on MSGs tissue sections (healthy control and patients with low, moderate, and severe lesions) to assess the presence and quantify *Notch2* mRNA using RNAscope probe sets. *Notch2* mRNA signal was observed in the epithelial cells of the acini and ducts, as well as in the interlobular infiltrative cells (Figure 3A–F). To understand the relationship between the severity of the inflammatory condition of the pSjD and the number of *Notch2* mRNA-positive cells in SGs biopsies, the *Notch2* mRNA expression was compared across the cell types observed in the SGs tissue, correlating it with the low, moderate, or severe inflammatory condition. In pSjD patients, there was a statistically significant positive correlation between the grade of inflammation and the percentage of acinar and ductal epithelial cells positive for *Notch2* mRNA ISH signals (Figure 3E). The mean percentage of *Notch2* mRNA-positive cells was 12 ± 0.93% to 86% ± 3.4% (from healthy to severe SjD) for acinar epithelial cells, and 8 ± 0.38% to 74% ± 2.3% for ductal epithelial cells (r = 0.76; *p* = 0.02; r = 0.91; *p* = 0.003, respectively); in addition, a significant correlation between *Notch2* mRNA expression in infiltrating inflammatory cells and severity of inflammation was also detected (16 ± 1.3% to 61% ± 6.7%) for infiltrating inflammatory cells from healthy to severe inflammatory grade (r = 0.34; *p* = 0.23) (Figure 3A–D). These results confirmed the presence of *Notch2* transcripts in acinar, ductal epithelial cells, and infiltrating inflammatory cells and that the level of *Notch2* mRNA detected in the SGs correlates with the severity of the inflammatory grade and could represent an index of evolving disease (Figure 3A–D).

### 3.3. Notch2 Protein Expression in SjD SGs Biopsies Was Correlated with the Degree of Inflammation

**Figure 4:**
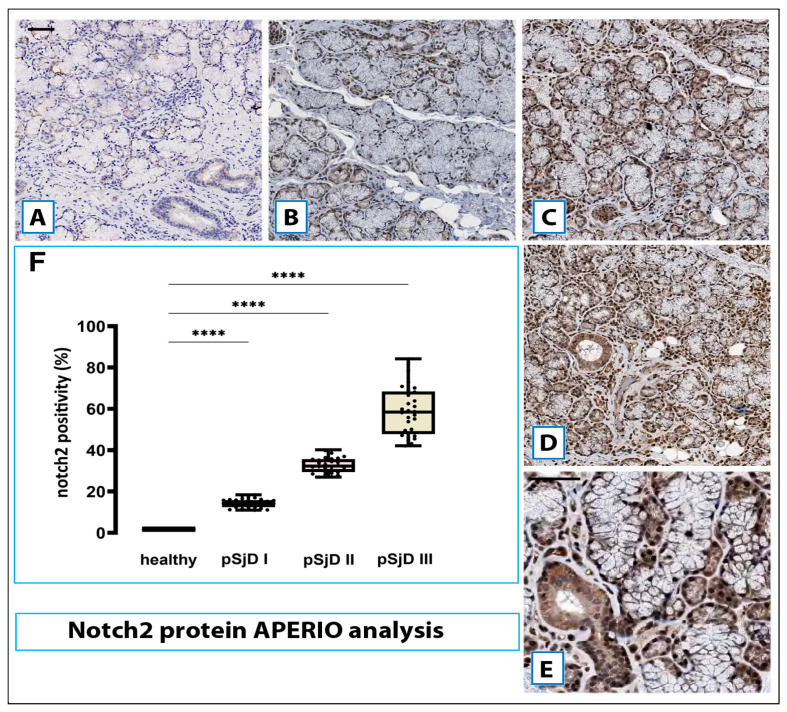
Notch2 protein detection in pSjD SGs tissues. Notch2 protein was detected by immunohistochemical analysis in pSjD SGs tissue sections of healthy control (**A**) and patients with different inflammatory grades (pSjDI, pSjDII, pSjDIII) (**B**–**E**). MSGs from healthy and pSjD biopsies were stained with primary antibodies against Notch2 and with the HRP-conjugated secondary antibody. The slides were incubated with diamino benzidine tetrahydrochloride as a substrate and counterstained with hematoxylin stain in blue. Image (**E**) represents the magnification of acini and ducts of pSjD III tissues. All images were scanned and analyzed with an Aperio ImageScope instrument. Image (**F**) represents the morphometric analysis of Notch2 protein in healthy and pSjD (I, II, III) biopsies expressed in terms of percent of signal positivity; The graph (**F**) demonstrates a significant increase of Notch2 protein in variously inflammatory grades of pSjD (**** *p* < 0 01) (data represent mean ± SE of three independent experiments). Bar = 20 μm.

Immunohistochemical analyses against Notch2 protein were performed, leading to results from three repeated experiments; two blinded, independent researchers worked. The expression level of Notch2 protein in the pSjD SGs tissue was shown in Figure 4. Notch2 protein immunoexpression had a fine speckled-punctuated pattern. In all the pSjD cases, immunostaining extended to more than 60% of ductal cells (grade III) and to more than 80% of acinar cells with moderate (grade II) or strong intensity (grade III), resulting in a “strongly positive” classification. The infiltrating cells showed a relevant positivity, which was found to be correlated with the increase in the subject’s inflammatory characteristics. All healthy subjects tested as controls were Notch2 negative. A statistically significant difference was found between the pSjD low, moderate, and severe grade of inflammation subgroups regarding the Notch2 immunoreactivity (*p* < 0.01).

As shown in Figure 4A–E, the positive Notch2 protein was predominantly located in the cell membrane and/or cytoplasm, especially in the acinar cells. Brown granular staining was deemed as positive performance.

**Figure 5:**
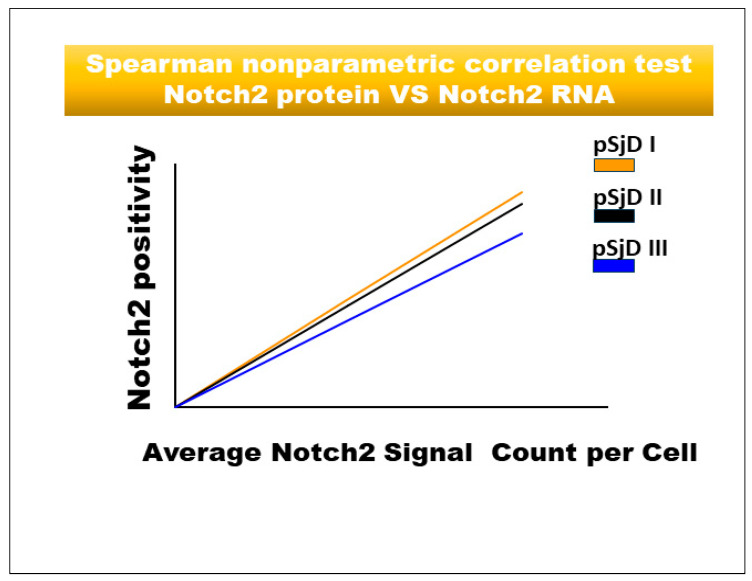
Spearman correlation graph between Notch2 protein and *Notch2* mRNA in different inflammatory grades of pSjD SGs tissue sections. Spearman test demonstrates a significant correlation between Notch2 protein expression and *Notch2* mRNA.

The normalized data pertaining to mRNA and protein expression were examined to ascertain mean values ± standard error (s.e.). The variations among the parameters were evaluated utilizing the Student’s *t*-test. Spearman’s correlation coefficients were calculated to evaluate the relationships between Notch2, ADAM17, and the pSjD inflammatory grade. A *p*-value of less than 0.05 was deemed statistically significant. In fact, a significant direct association between acinar cellular staining for Notch2 and the histological inflammatory grade of pSS was reported (r = 0.571 for grade I, r = 0.678 for grade II, and r = 0.786 for grade III; *p* < 0.05). Similarly, high Notch2 expression was closely associated with a high inflammatory degree in ductal cells (r = 0.64 for grade I, r = 0.76 for grade II, and r = 0.92 for grade III; *p* < 0.05). Similarly, investigating the expression of Notch2 in infiltrating lymphocytes in association with the patients’ inflammatory condition with Spearman’s correlation analysis, a positive correlation was also demonstrated (r = 0.345 for grade I, r = 0.402 for grade II, and r = 0.715 for grade III; *p* < 0.001). Similarly, when the correlation between the expression of Notch mRNA and Notch protein was statistically examined, a significant positive correlation ratio was observed for each inflammatory grade (r = 0.74 for grade I, r = 0.72 for grade II, and r = 0.92 for grade III; *p* < 0.05, *p* < 0.05) (Figure 5).

### 3.4. Notch2 Expression Correlates with ADAM17 Expression in SjD SGs Tissue

**Figure 6:**
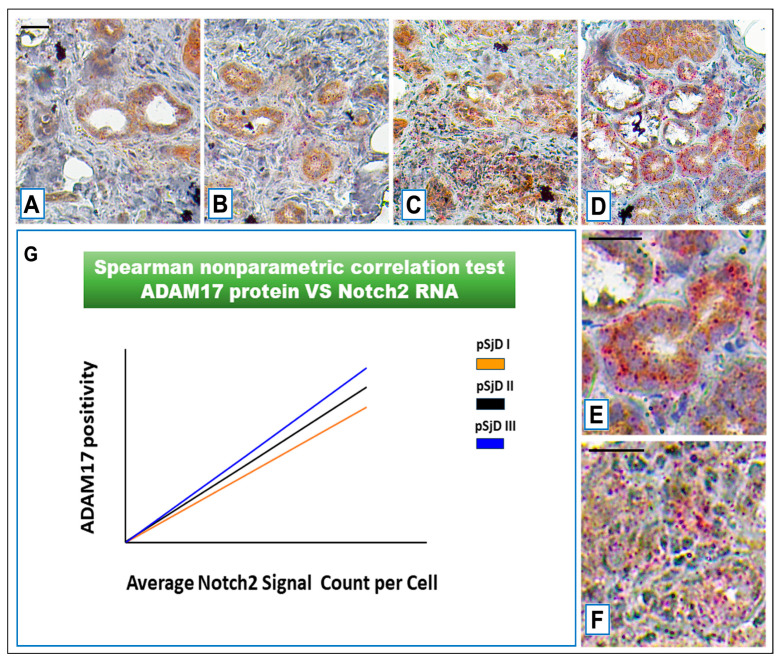
*Notch2* RNA scope and ADAM17 protein detection in pSjD SGs tissue sections of healthy control (**A**) and patients to different inflammatory grades (pSjDI, pSjDII, pSjDIII) (**B**–**D**). Images (**E**,**F**) represent the magnification of acini and ducts of pSjD III tissues. *Notch2* mRNA was analyzed by in an in situ hybridization technique, and ADAM17 protein was detected by immunohistochemical analysis in pSjD SGs tissue sections of healthy control (**A**) and patients of different inflammatory grades (pSjDI, pSjDII, pSjDIII) (**B**–**E**). Image (**E**) represents the magnification of acini and ducts of pSjD III tissues. Image (**G**) represents the graph of correlation between *Notch2* mRNA and ADAM17 protein. The Spearman non-parametric correlation test demonstrates a significant correlation between ADAM17 protein expression and *Notch2* mRNA. All images were scanned and analyzed with an Aperio ImageScope instrument. Bar = 20 μm.

To find evidence for an association between *Notch2* mRNA and ADAM17 expression in MSGs of pSjD patients, some bioptic specimens, including the appropriate healthy controls (see Section 2 for details), were disassembled and used for IHC analysis. We examined the expression of ADAM17 by IHC in pSjD biopsy samples in comparison with healthy subjects. The number and the distribution of lymphocytic foci in the different pSjD SGs bioptic specimens allowed us to classify the samples as I, low; II, intermediate; and III, advanced, respectively, as reported in Section 2. Ten biopsy specimens for each group were analyzed. Anti-human ADAM17 antibody mAb was used to evidence SGs ADAM17 expression (Figure 6A–F). As shown in Figure 6, strikingly, all pSjD SGs samples revealed a positive staining for ADAM17, whereas normal tissues showed very low ADAM17 expression (*p* < 0.01). The staining of ADAM17 protein ranged from weak to strong, moving from low to high inflammatory levels (*p* < 0.01), and the results clearly showed that ADAM17-positive staining was increased in those biopsies, characterized by a higher inflammatory degree that are also characterized by elevated expression of Notch2 mRNA expression. As observed, in all pSjD glandular specimens, ADAM17 seems to be expressed both in acinar, ductal cells, and in infiltrating lymphocytes (Figure 6A–D); interestingly, ADAM17’s expression is correlated with increased immune cell infiltration. Very important is the observation of a positive correlation between ADAM17 expression and Notch2 mRNA expression within all 30 pSjD analyzed SGs samples (r = 0.88 for grade I, r = 0.83 for grade II and r = 0.71 for grade III; *p* < 0.05) (Figure 6G).

**Figure 7:**
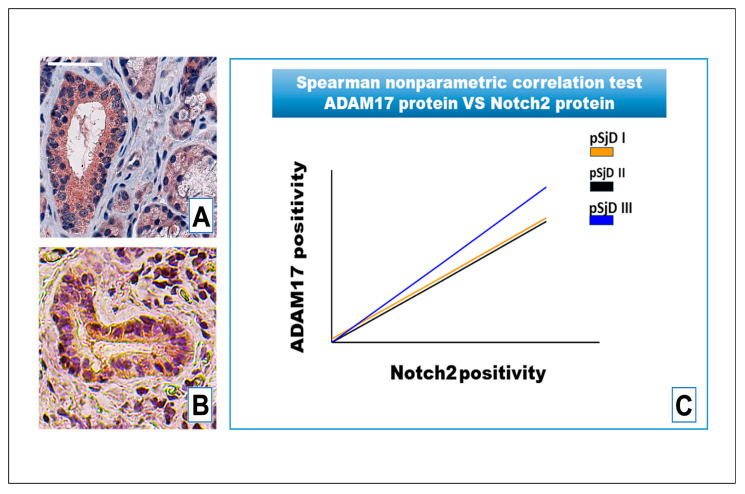
IHC analysis of Notch2 protein and ADAM17 protein detection in pSjD SGs tissue sections with inflammatory grade III (**A**,**B**). Image (**C**) visualizes Spearman correlation graph between Notch2 protein and ADAM17 protein in different inflammatory grades of pSjD SGs tissue sections. Correlation test demonstrates a significant correlation between ADAM17 protein and Notch2 protein. Bar = 20 μm.

Importantly, ADAM17 staining also correlated with protein levels of Notch2 in SjD samples (r = 0.74 for grade I, r = 0.7 for grade II and r = 0.92 for grade III; *p* < 0.05) (Figure 7A–C).

## 4. Discussion

Notch signaling is physiologically important for cell-to-cell communication and for controlling multiple cell differentiation processes during embryonic and adult life [25]. Notch2, in particular, is described to reveal activity in liver, kidney, ovary, smooth muscle, and T and B lymphocyte development [26,27].

Nevertheless, there are many controversies concerning Notch2’s role in pathological conditions, particularly in autoimmune diseases, characterized by sustained and chronic inflammation.

Conversely, the role of ADAM17 has been extensively evaluated in several autoimmune diseases, and this molecule has been shown to be located at the crossroads of various molecular pathways involved in the transcription and translation of pro-inflammatory factors. The role of ADAM17 has also been extensively evaluated in the chronic inflammatory disease SjD, the subject of the experimental project presented in this work. In SjD, ADAM17 activation has been correlated with the overexpression of inflammatory molecules responsible for sustained chronic inflammation and implied in the generation of fibrotic tissue in the SGs [28].

Inflammation is, in fact, associated with a wide range of diseases, including asthma, arthritis, cancer, obesity, heart disease, colitis, neurological disorders, and autoimmune diseases. Detection of involved factors and analysis of their receptors, as well as investigation of the pathways activated, are critical for understanding, and subsequently, individualizing a treatment for many inflammatory diseases. In SjD, ADAM17 is able to activate the Amphiregulin/Epidermal growth factor receptor (EGFR) pathway and the VEGF-A/VEGFR2/NF-κB axis, whose dysfunction may be contributory to the pathogenesis and exacerbation of SjD [29].

An in-depth analysis of the role of ADAM17 in SjD has not yet been matched, to date, by an equally comprehensive analysis of the expression of Notch2 in SjD, nor has there been investigated a possible correlation between the cellular expression of Notch2 and ADAM17 or a possible correlation between the expression of these two closely interconnected factors and the degree of inflammation observed in SGs of SjD patients.

In SjD recent research has demonstrated an increase in *Notch2* mRNA expression in B cells located in the marginal zone of the SGs; Notch mRNA was also detected in the germinal centers of tonsil biopsies from patients with pSjD [30]. Elevated Notch2 and PR domain zinc finger protein 1 mRNA levels and increased B-lymphocyte induced-maturation protein 1 (BLIMP-1) mRNA expression were demonstrated within clusters of transient type II B cells present in the SGs from pSjD patients [31]. Furthermore, molecular investigations performed at a clinical level have demonstrated a clear correlation between the risk of developing MALT, the main type of SGs lymphoma found in SjD patients, and mutations in the *Notch2* gene [32]. This seems to support a correlation between lymphoma susceptibility and Notch activation in pSjD [33]. In addition, given the recent discovery of a lively cross-talk between Notch and Wnt signaling during organ regeneration [34], in recent years, the possibility of a defective dialogue between Wnt and Notch at the basis of the onset and/or pathogenesis of SjD has been evaluated. But the most fascinating challenge is to find the key that could lead to the simultaneous activation of various inflammatory pathways, which has been demonstrated in pSjD, leading to the identification of a dysregulation of NF-κB responsible for an anomalous activation of Notch, correlated to the activation of the Hippo-mediated transduction cascade [35]. Also, a wrong communication mechanism between glandular epithelial cells and stromal cells was identified as the basis of the alteration of the structure of the SGs observed in SjD, which, once again, appears to be mediated by Notch activation. Indeed, the expression of specific genes in stromal cells is regulated by molecules released by epithelial cells in patients with SjD; in particular, the release of factors implicated in the Notch signaling pathway, such as Notch2/3, appears to depend on epithelial-derived MDK and SCGB3A1, which play a role in promoting fibroblast transformation and generating fibrosis [36].

Altered Notch signaling also appears to be involved in characteristic systemic manifestations in SjD, characterized by widespread pain probably triggered through the activation of TLR signaling [31]. Through the activation of TLR, Notch signaling determines the activation and proliferation of macrophages and dendritic cells and the production of inflammatory cytokines, such as TNF-α and IL-1β. Inhibiting the signaling pathways that involve the activation of Notch with a specific inhibitor in these patients can prevent the onset of neuropathies, confirming the involvement of Notch in the perception of pain associated with SjD [37].

Furthermore, Notch2 altered expression could be an early disease marker because increased Notch2 signaling is observed during the early, asymptomatic stages of SjD, particularly in SGs [37], and Notch2 could be involved in the inflammatory response since Notch2 signaling contributes to a sustained type 1 interferon (IFN) production, a key feature of inflammation in SjD [38].

Now, on the basis of these premises, in designing the experimental phase of this work, we asked ourselves the following question: if ADAM17 has multiple roles in the pathogenesis of SjD and Notch2 is widely considered a key player in various inflammatory mechanisms in SjD, and, in addition, ADAM17 is known to activate Notch signaling, could there be a correlated expression of these two factors in SGs of pSjD patients, and could the regulation of their expression be related to the degree of inflammation in patients?

To demonstrate a possible correlated expression between Notch2 and ADAM17, and the grade of inflammatory conditions in pSjD, we used, in this work, the combination of mRNA analysis via ISH and protein analysis via IHC in the same section; this extremely powerful technique allows us to study, in a more in-depth manner, the expression of the *Notch2* gene and protein in biopsies of SjD patients with different degrees of inflammation and to evaluate a probable correlation between an increase in the expression of Notch2 and ADAM17 in SjD, overcoming inevitable limitations deriving from the use of other methods.

This methodological approach allowed us to demonstrate a clear correlated overexpression of Notch2 and ADAM17 in SGs biopsy specimens derived from pSjD patients; the expression of both factors significantly increases accordingly with the inflammatory grade in acinar cells, ductal cells, lymphocytic infiltrates, and myoepithelial cells. We hope that this further piece added through our work to the elucidation of the pathways involving ADAM17 and Notch2 may be of help in translational studies for the identification of effective therapies for pSjD.

## Figures and Tables

**Table 1:** Main characteristics of patients with pSjD engaged in this study and relative healthy controls.

Domain	Healthy	pSjD I	pSjD II	pSjD III
Age at diagnosis, years	49.8 (range 21–66)	57.7 (range 24.6–66.9)	52.4 (range 24.8–66.7)	52.9 (range 38.9–70.1)
Sex	female	female	female	female
Focus scores	negative	low lesions (FS ≤ 1)	moderate lesions (FS ≥ 2)	severe lesions FS ≥ 2 and GC+
Anti-Ro positivity n (%)	negative	positive	positive	positive
Schirmer test ≤ 5 mm/5 min in at least 1 eye	negative	positive	positive	positive
**Clinical parameters**				
Unstimulated salivary flow (mL/5 min)	negative	mild hypofunction (>0.7 mL/min)	moderate hypofunction (0.1 to 0.7 mL/min)	severe hypofunction (>0.1 mL/min)
Rheumatoid factor	negative	positive	positive	positive
Anti-SSA antibody	negative	positive	positive	positive
Anti-SSB antibody	negative	positive	positive	positive
Anti-RNP antibody	negative	positive	positive	positive
Anti-centromere antibody	negative	negative	positive	positive
Anti-DNA antibody	negative	negative	negative	negative
**Patient-reported outcomes**				
Pain	negative	absence	presence	presence
Fatigue	negative	absence	presence	presence
Overall dryness	ocular and oral dryness	ocular and oral dryness	ocular and oral dryness	ocular and oral dryness
**Systemic manifestations according to ESSDAI domains**				
Glandular	normal salivary glands	unilateral salivary gland enlargement	swelling of mostly the parotid gland	swelling of mostly the parotid gland; diffuse sialectasis
Articular	absence of articular events	absence	arthralgia; arthritis	arthralgia; arthritis
Muscular	absence of muscular pains	muscle weakness	muscle weakness; myositis	myalgia; muscle weakness; myositis
Renal	absence	absence	absence	nephrogenic diabetes insipidus; proximal tubular acidosis; hypokalemia
Peripherical nervous system	absence	absence of neuronal pathological events	painful in the distal extremities; radiculoneuropathy; autonomic neuropathy	sensory ataxic neuropathy; cranial neuropathies; radiculoneuropathy;
Central nervous system	absence	absence of neuronal pathological events	motor or sensory deficits	spinal cord involvement cognitive dysfunction
Lymphadenopathy	absence	absence	splenomegaly	splenomegaly

## Data Availability

The original contributions presented in this study are included in the article. Further inquiries can be directed to the corresponding author.
